# A single-center retrospective safety analysis of cyclin-dependent kinase 4/6 inhibitors concurrent with radiation therapy in metastatic breast cancer patients

**DOI:** 10.1038/s41598-020-70430-2

**Published:** 2020-08-12

**Authors:** Andrea Emanuele Guerini, Sara Pedretti, Emiliano Salah, Edda Lucia Simoncini, Marta Maddalo, Ludovica Pegurri, Rebecca Pedersini, Lucia Vassalli, Nadia Pasinetti, Gloria Peretto, Luca Triggiani, Gianluca Costantino, Vanessa Figlia, Filippo Alongi, Stefano Maria Magrini, Michela Buglione

**Affiliations:** 1 Department of Radiation Oncology, Brescia University, Piazzale Spedali Civili, 1, 25123 Brescia, Italy; 2 Department of Radiation Oncology, ASST Spedali Civili of Brescia, P.le Spedali Civili 1, 25123 Brescia, Italy; 3 Medical Oncology Unit, University of Brescia, Spedali Civili of Brescia, P.le Spedali Civili 1, 25123 Brescia, Italy; 4 Radiation Oncology Service, ASST Valcamonica, 25040 Esine, Italy; 5 Advanced Radiation Oncology Department, Cancer Care Center, IRCCS Sacro Cuore Don Calabria Hospital, Via Don Sempreboni 5, 37034 Verona, Negrar Italy

**Keywords:** Breast cancer, Cancer therapy

## Abstract

Cyclin dependent kinases 4/6 (CDK4/6) inhibitors gained an essential role in the treatment of metastatic breast cancer. Nevertheless, data regarding their use in combination with radiotherapy are still scarce. We performed a retrospective preliminary analysis of breast cancer patients treated at our Center with palliative radiation therapy and concurrent CDK4/6 inhibitors. Toxicities were measured according to CTCAE 4.0, local response according to RECIST 1.1 or PERCIST 1.0 and pain control using verbal numeric scale. 18 patients (32 treated sites) were identified; 50% received palbociclib, 33.3% ribociclib and 16.7% abemacliclib. Acute non-hematologic toxicity was fair, with the only exception of a patient who developed G3 ileitis. During 3 months following RT, 61.1% of patients developed G 3–4 neutropenia; nevertheless no patient required permanent suspension of treatment. Pain control was complete in 88.2% of patients three months after radiotherapy; 94.4% of patients achieved and maintained local control of disease. Radiotherapy concomitant to CDK4/6 inhibitors is feasible and characterized by a fair toxicity profile, with isolated episodes of high-grade reversible intestinal toxicity. Rate of G 3–4 neutropenia was comparable with that measured for CDK4/6 inhibitors alone. Promising results were reported in terms of pain relief and local control of disease.

## Introduction

Selective cyclin dependent kinases 4/6 (CDK4/6) inhibitors block tumor suppressor retinoblastoma protein phosphorylation, preventing the transition of cancer cells from G1 to S phase with consequent inhibition of cell cycle and proliferation^1^. To date, three CDK4/6 inhibitors are approved against hormone receptor positive, human epidermal growth factor receptor 2 negative metastatic or advanced breast cancer in combination with aromatase inhibitors or fulvestrant both in first and subsequent lines of therapy. After different phase III trials^2–7^ reporting significant improvements in response rate and progression free survival, palbociclib was the first CDK4/6 inhibitor authorized by European Medicines Agency in November 2016, followed by ribociclib in August 2017 and abemaciclib in September 2018.

The three compounds demonstrated comparable outcomes in terms of antitumoral efficacy, but are characterized by substantial differences in pharmacokinetics and few discrepancies in toxicity profiles^8^. Palbociclib and ribociclib showed a predominant bone marrow toxicity, with G 3–4 neutropenia reported in up to 66.7% of patients^3^. Abemaciclib determined lower rates of neutropenia, but a higher frequence of G 3–4 diarrhea (up to 19.7% compared to up to 4% for palbociclib)^2,9^. Ribociclib also determined G 3–4 aspartate aminotransferase and alanine aminotransferase elevations in 5–10% and QT interval prolongation in ~ 1–3% of treated patients^4,5^. Radiotherapy has a central and established role in the palliation of symptomatic lesions in metastatic cancer^10^ and is emerging as a mean to improve local control and prognosis of oligo-metastatic patients^11,12^. Despite the wide use of CDK4/6 inhibitors in the treatment of breast cancer, published data regarding possible contraindications and interactions with radiation treatment are still very limited. This, combined with isolated case reports^13,14^ of high grade radio-induced toxicity could raise concern in many clinicians and lead them to avoid the combination with radiotherapy in fear of an increase in toxicity and thus deprive the patient of an effective treatment.

We therefore performed a retrospective preliminary analysis of breast cancer patients treated at our Center with palliative radiation therapy to bone lesions and concurrent CDK4/6 inhibitors to assess the possible pitfalls of this combination.

## Methods and materials

### Study population

We retrospectively reviewed the records of all patients affected by metastatic breast cancer that received external beam radiation therapy at our Center (Brescia University Radiation Therapy Department) from 2016 to 2020. Patients who were treated with CDK4/6 inhibitors concomitantly with palliative radiotherapy were included in this analysis: the maximum interval allowed between last drug administration and radiotherapy was 2 half lives (about 58 h for palbociclib^15^, 64 h for ribociclib^16^ and 37 h for abemaciclib^17^). Most of the patients underwent systemic treatment at Brescia University Oncology Department, in the context of our institutional Breast Unit.

### Systemic treatment

Palbociclib was prescribed at the dose of 125 mg daily for 21 days followed by 7 days of pause, in association with either letrozole 2.5 mg daily or fulvestrant 500 mg every 28 days. Ribociclib was prescribed at the dose of 600 mg daily from day 1 to day 21 every 28 days, in combination with daily letrozole. Abemaciclib was prescribed at the dose of 150 mg bis in die, in association with either letrozole 2.5 mg daily or fulvestrant 500 mg every 28 days. Premenopausal patients also received LHRH agonists. Dosage reductions were allowed at prescriber’s discretion on the base of hematologic and clinical toxicities.

### Radiation therapy

Most of the treatments (30 of 32) were prescribed with symptomatic or palliative intent, planned with 3D conformal techniques and patients were treated with 6 or 10 MV beams, generated from Elekta Synergy® linear accelerator. Two treatments for oligometastatic disease were prescribed with ablative intent, planned with stereotactic techniques one using VMAT and one using Tomotherapy and erogated respectively with Elekta Synergy® and Tomotherapy Hi-ART® systems. Clinical target volume (CTV) was defined on a case-by-case basis, generally including the osteolytic lesion and the eventual extension to the adjacent soft-tissues with a margin of 1–3 cm corrected for anatomical structures. For spinal lesions, the CTV usually included the involved vertebra plus half of the upper and lower vertebrae. Planning target volume (PTV) was generally outlined adding a 9 mm margin to the CTV. Prescribed dose was chosen according to guidelines for palliative radiotherapy^18,19^ and tailored to the characteristics of the patient, the tumor burden and the location of the lesion; in all the plans 95% of prescribed dose covered at least 95% of the PTV.

### Outcome measures and statistics

Toxicities were graded using National Cancer Institute Common Terminology Criteria for Adverse Events version 4.0 (CTCAE 4.0). Tumor response was determined using CT scans, MRI or 18FDG-PET-CT scans and defined according to Response Evaluation Criteria in Solid Tumors 1.1 (RECIST 1.1) or Positron Emission Tomography Response Criteria in Solid Tumors (PERCIST 1.0). Pain intensity was assessed by the patient’s self-rated pain verbal numeric scale (VNS), in which patients are asked to verbally state a number between 0 (no pain) and 10 (worst imaginable pain).

### Ethical approval

All the procedures performed were in accordance with the ethical standards of the institutional and/or national research committee and with the 1964 Helsinki declaration and its later amendments or comparable ethical standards. Informed consent, when required according to institutional, national and international laws, was obtained from all subjects. This study was approved by the Ethics Committee of our Institution (ASST Spedali Civili di Brescia).

### Disclaimer

This manuscript has not been published and is not under consideration for publication elsewhere.

## Results

### Study population

Eighteen metastatic breast cancer patients were selected, with a total of 32 treated sites. Baseline patients’ characteristics are detailed in Table 1. Median age at RT start was 64 years, four patients were metastatic at diagnosis and median interval between first diagnosis and metastatic progression was 6.8 years. Over 70% of the patients had not previously received endocrine therapy for metastatic disease. Nine patients (50%) received palbociclib, six (33.3%) ribociclib and three (16.7%) abemacliclib (concomitant RT on 19, 10 and 3 disease sites respectively). Most of the patients (66.7%) had both visceral and bone disease, while 33.3% presented with only osseous involvement. Concomitant hormone-therapy was represented by letrozole for 44.4% and fulvestrant for 55.6% of patients. Median and mean interval between CDK4/6 inhibitor start and RT were 1.5 and 3.2 months.

**Table 1:** Main patients' characteristics.

**Median age**	
Diagnosis	50.5 y (range 31.6–77.3 y)
Metastasis	60.9 y (range 35.8–77.3 y)
RT start	64.1 y (range 36.1–77.5 y)
Time from diagnosis to metastasis	6.8 y (range 0–24.4 y)
**Histology**	
Ductal/NST carcinoma	11 (72.2%)
Lobular carcinoma	2 (11.1%)
Micropapillary	1 (5.6%)
Mixed ductal and lobular	1 (5.6%)
Unknown	1 (5.6%)
**Metastatic at diagnosis**	
Yes	4 (22.2%)
No	14 (77.8%)
**Site of metastic disease**	
Bone only	6 (33.3%)
Bone and visceral	12 (66.7%)
**Previous hormonal treatment for metastatic disease**	
Yes	5 (27.8%)
No	13 (72.2%)
**CDK4/6 inhibitor**	
Palbociclib	9 (50%)
Ribociclib	6 (33.3%)
Abemaciclib	3 (16.7%)
**Concomitant hormonal treatment**	
Fulvestrant	10 (55.6%)
Letrozole	8 (44.4%)

### Treatment characteristics

Details regarding radiation therapy are summarized in Tables 2 and 3. All the patient received radiation therapy to bone metastatic sites including the spine (61.1% of patients), the pelvis (50%), extremities (22.1%), sternum (11.1%), skull (5.6%) or ribs (5.6%). Prescribed dose was 30 Gy in 10 fractions for 40.6% of treatments, 20 Gy in 5 fractions for 37.5%, 8 Gy single fraction for 15.6% and 30 Gy in 3 fractions for 6.3%. The majority of treatments were performed with 3D conformal radiotherapy (90.6%), while two were calculated with VMAT and one with Tomotherapy.

**Table 2:** Radiotherapy treatments characteristics.

Time from CDK4/6 start to RT	
Median 1.5 months	Range 0–15. 6 months
**Total number of treated sites**	
32	Range 1–4 per patient
**Treatment dose/fractions**	
30 Gy/10 fr	13 (40.6%)
20 Gy/5 fr	12 (37.5%)
8 Gy/1 fr	5 (15.6%)
30 Gy/3 fr	2 (6.3%)
**RT technique**	
3D-CRT	29 (90.6%)
VMAT	2 (6.3%)
Tomotherapy	1 (3.1%)
**Treated site**	
Spine	61.1% of patients
Pelvis	50%
Extremities	22.2%
Sternum	11.1%
Skull	5.6%
Ribs	5.6%

**Table 3:** site and characteristics of radiotherapy treatment and pain and radiological response.

Patient	RT site	Concomitant CDK4/6 inhibitor	RT dose/technique	VNS before RT	VNS at RT end	VNS at 1 mo	Response at 3 mo	Response at 6 mo	LR	Acute toxicity
1	L5 + sacrum + R ilium	Palbociclib	30 Gy/10 fr—3DCRT	6	4	3	SD	SD	No	G3 ileitis
2	L5 + sacrum + R sacroiliac joint	Palbociclib	30 Gy/10 fr—3DCRT	4	4	3		PR	No	G1 diarrhea
3	L femur head; L IX rib	Palbociclib	20 Gy/5 fr—3DCRT	0	0	0		PR	No	None
4	R ischium + R ilium + R femur head + S2; C4; T2	Palbociclib	30 Gy/10 fr—3DCRT	7	5	0	PR	PR	No	G1 sore-throat + G1 diarrhea
5	R ilium + R sacral ala	Palbociclib	20 Gy/5 fr—3DCRT	2	1	0	PR	PR	Yes	None
6	L acetabulum + L femur head; R sacral ala C5 vertebra	Palbociclib	30 Gy/10 fr—3DCRT 30 Gy/3 fr—Tomo	8 0	5 0	0 0		CR PR	No No	G1 nausea + G1 fatigue None
7	Sacrum + R ilium + bilateral femur head	Palbociclib	20 Gy/5 fr—VMAT	7	4	2			No	None
8	T7 vertebra; T12 vertebra; S3-S5 tract	Ribociclib	30 Gy/5 fr—3DCRT	0	0	0	SD	PR	No	G1 nausea
9	T6 vertebra	Ribociclib	20 Gy/5 fr—3DCRT	5	3	0		SD	No	G1 fatigue
10	T2-T4 vertebral tract	Palbociclib	20 Gy/5 fr—3DCRT	6	6	0	SD		No	None
11	T4 vertebra; T9 vertebra; R acetabulum	Ribociclib	20 Gy/5 fr—3DCRT	3	0	0	SD	PR	No	G1 esophagitis
12	T2 vertebra	Palbociclib	20 Gy/5 fr—3DCRT	0	0	0	PR	PR	No	None
13	L ischium + L pubic bone	Ribociclib	8 Gy/1 fr—3DCRT	0	0	0	PR		No	None
14	sternum	Ribociclib	8 Gy/1 fr—3DCRT	2	2	0			No	None
15	T4 vertebra	Abemaciclib	8 Gy/1 fr—3DCRT	0	0	0	PR		No	None
16	L femur	Ribociclib	8 Gy/1 fr—3DCRT	4	4	0	SD	PR	No	None
17	T10-L1 vertebral tract	Abemaciclib	8 Gy/1 fr—3DCRT	5	5	0			No	None
18	sternum	Abemaciclib	30 Gy/3 fr—VMAT	0	0	0			No	None

VNS, pain verbal numeric scale; mo, months; LR, local recurrence; L, left; R, right; fr, fraction; 3DCRT, 3D conformal radiotherapy; Tomo, Tomotherapy; VMAT, Volumetric Modulated Arc Therapy; SD, stable disease; PR, partial response.

### Toxicity

Acute toxicity data are summarized in Table 3. All radiotherapy courses were administered as planned without suspensions or dose reduction. Acute toxicity was limited and represented mainly by grade 1 side effects including sore throat (11.1% of patients), nausea/vomit (11.1%), diarrhea (11.1%), fatigue (11.1%) and esophagitis (5.6%).

The only exception was represented by one case of gastrointestinal G3 toxicity: 10 days after the end of RT (30 Gy in 10 fractions on a bulky bone localization involving L5 vertebra, sacrum and right ischium) the patient developed worsening diarrhea (up to 10 stools/day) and abdominal pain, that required hospitalization. A CT scan showed wall thickening and luminal narrowing of the distal ileum and colonoscopy confirmed the diagnosis of radiation-induced ileitis. Following conservative management with antibiotics and anti-inflammatory drugs, the toxicity completely resolved after 20 days. An abdominal CT-scan performed 3 months later confirmed the complete radiological resolution of ileitis. Palbociclib was suspended for a cycle and later resumed at full dosage and is still ongoing 29 months after this episode; dosage was reduced at 100 mg/die for G3 neutropenia more than 12 months after RT and subsequent isolated episodes of grade 1 diarrhea were reported. The patient later underwent stereotactic radiotherapy (30 Gy/3 fractions) on C5 vertebra: during this treatment, palbociclib was suspended to avoid excessive toxicity. Dosimetric parameters of the eight patients treated on pelvic sites are reported in Table 4. It has to be noted that the other seven patients treated to high-volume pelvic sites with similar dosimetric parameters did not develop high grade intestinal toxicity.

**Table 4:** Dosimetric parameters of the eight patients treated on pelvic sites.

Patient	CTV (cc)	PTV (cc)	Intestinal Dmean (Gy)	Intestinal Dmax (Gy)	Intestinal D50 (Gy)	Intestinal V10 (%)	Dose/fraction	Diarrhea
Pt 1: L5 + sacrum + R ilium	944.5	1853.9	10	31	16.7	37	30 Gy/10fr EQD2 32.5	G 3
Pt 2: L5 + sacrum + R sacroiliac joint	545	1,138.9	11.2	30.9	9.2	45	30 Gy/10fr EQD2 32.5	G 1
Pt 4: R ischium + R ilium + S2	491.8	1,053.1	7.7	31.2	2.8	30	30 Gy/10fr EQD2 32.5	G 1
Pt 5: R ilium + R sacral ala	151.7	232.1	1.5	20	0.2	5	20 Gy/5 fr EQD2 23.3	No
Pt 6: L acetabulum + R sacral ala	257.4	666.1	6.7	31.2	1.3	27	30 Gy/10fr EQD2 32.5	No
Pt 7: Sacrum + R ilium	933.7	1819.4					30 Gy/10fr EQD2 32.5	No
Pt 8: S3-S5 tract	36.1	109.7	2.6	30.3	0.2	6	30 Gy/10fr EQD2 32.5	No
Pt 13: L ischium + L pubic bone	89.6	214.9	2.5	8	1.9	0	8 Gy/1fr EQD2 12	No

Only one patient (5.6%) experienced “pain flare” during radiotherapy, that resolved before the end of treatment with anti-inflammatory drugs. Neutropenia was developed by 88.8% of the patients during 3 months following RT and was grade 3–4 in 61.1% of patients. Due to such toxicity, during the 3 months following RT CDK4/6 inhibitor was temporary suspended in 57.1% of the 14 patients with available data and the dose was temporary reduced in 28.6% and permanently reduced in 14.3% of patients; no permanent withhold was required and the median duration of drug suspension was 7 days (range 7–21 days). It has to be noted that all the four patients that were already on treatment with CDK4/6 inhibitors for more than three months before RT start already reported G ≥ 3 neutropenia during the previous cycles. Long term non-hematologic toxicity (median follow up 12 months) included G1 nausea/vomit (11.1% of patients), G1 diarrhea (11.1%) and G1 infections (5.6%).

### Treatment Outcome

Median VNS of the 12 patients presenting with pain was 5 (range 2–8) before RT and 4 (range 0–6) at the last day of RT; pain relief was complete in 8.3% and partial in 50% of patients, while the remaining 41.7% reported stable pain. One month after RT 83.3% of patients reported complete pain control and median VNS was 0 (mean 0.6 for previously symptomatic patients and 0.4 for the whole population) and after 3 and 6 months control was complete in 88.9% of patients and median VNS was 0 (mean 0.5 and 0.3 respectively). Only two patients still experienced pain after one and three months, both with a VNS reduction of at least 50% compared to baseline. No pain recurrence was reported. With a median follow up of 13.7 months (range 2–29 months) 15 patients (83.3%) are still alive, 6 (33.3%) developed progressive disease (resulting in 3 deaths) and the other 12 (66.7%) did not and are still on treatment with CDK4/6 inhibitors. Radiological and/or metabolic local response was evaluable for 11 patients at three months after RT (54.5% partial response, 45.5% stable disease) and for 12 patients after 6 months (8.3% complete response, 75% partial response and 16.7% stable disease compared to baseline). Considering the whole follow up period, 17 patients (94.4%) achieved local control of disease and maintained it until last evaluation and only one patient faced local recurrence 8 months after RT (20 Gy in 5 fractions to the right ilium and ala of sacrum).

## Discussion

Bone represents the most frequent site of distant dissemination from breast cancer, as up to about 13% of patients diagnosed stage I-III disease develop bone metastasis, considering a median follow up of 12.5 years^20^. Complications depend on the site and the characteristics (osteolytic, osteoplastic or mixed) of bone lesions and include loss of structural function, fractures, spinal cord compression and pain. Up to 75% of the patients bearing bone metastases experience invalidating pain^21^. Radiotherapy provides pain relief to 50–85% of patients, with up to one third obtaining complete response^22^. Pain improvement is usually achieved within one month after RT, and mean duration of pain control is approximately 19 weeks, or even longer in breast cancer patients^23^. Unfortunately, a consistent proportion of patients might not obtain pain relief after radiotherapy and about half of initial responders experience pain relapse within 12 months after treatment^24^. The concurrent administration of a drug with radio-sensitizing activity could therefore be a valuable mean to improve symptomatic and disease control. CDK4/6 inhibitors demonstrated radio-sensitizing effects not only on breast cancer^25^, but also on several other tumor cell lines, including colorectal carcinoma^25^, lung cancer^25,26^, atypical teratoid rhabdoid tumor^27^, glioblastoma^27,28^, hepatocellular carcinoma^29^, cholangiocarcinoma^29^ and head and neck squamous carcinoma^30^. Despite these promising activity, the lack of a substantial body of clinical literature might persuade clinicians to avoid the concomitant administration of RT and CDK4/6 inhibitors, as radio-sensitizing effect could potentially involve also healthy tissues and lead to increased toxicity. This might determine unnecessary interruption of systemic treatment or even abstention from the benefits of RT. Therefore, we performed a retrospective analysis of the patient that underwent RT with concomitant CDK4/6 inhibitors at our Institution and a review of previously published clinical literature. The first reports of the combination of RT with CDK4/6 inhibitors are represented by preliminary data from very small patient samples.

Hans et. al. described an acceptable toxicity profile in five metastatic breast cancer patients treated with palbociclib and concurrent palliative RT^31^; all the patients experienced symptom improvement, but follow up time and local control were not reported. Meattini et al. analyzed ribociclib concomitant to palliative RT for bone metastases in the first five patients treated at their institution^32^. Two patients developed grade 3–4 side effects (one neutropenia and one vomit and diarrhea) and two needed temporary suspension of ribociclib, but radiotherapy was never suspended. At 3-month assessment, three stable disease and two partial response were observed. Larger subsequent series provided a broader perspective regarding the safety of concomitant treatment with CDK4/6 inhibitors and radiotherapy. Chowdary et al. evaluated the safety and efficacy of palbociclib in 16 breast cancer patients that received RT for symptomatic metastases^33^. No significant toxicity increase was observed compared to rates reported for palbociclib alone; prolonged pain control was achieved in all patients, and no local failures were described. It has to be noted that only 31.3% of the patients received palbociclib concomitantly, as an interval of 14 days between drug administration (before or after) RT was allowed, with a median interval of 5 days. Ippolito et. al. analyzed 16 metastatic breast cancer patients (24 radiotherapy treatments) that received palbociclib or ribociclib concurrently with RT^34^. Most of those patients (68.7%) underwent palliative RT to bone lesions with a median dose of 30 Gy, while the remaining received higher doses (median 50 Gy) to oligo-metastatic or oligo-progressive sites. Neutropenia was common but was already reported in previous cycles for most of the patients, thus radiotherapy did not appear to determine a relevant myelo-suppressive effect. No other relevant toxicities were developed. Pain relief was obtained by all patients that received RT on bone metastases. In patients treated at higher doses two complete response were observed (patient with extra-bone disease), while two partial responses and one stable disease were reported in patients with osseous lesions. The main limits of this study are represented by the short median follow up (only 6.3 months) and the fact that long term toxicities were not analyzed and local disease control was reported only for the five oligo-metastatic and oligo-progressive patients. The toxicities observed for combined treatment in these series and in our study are in line with published safety data of patients treated with CDK4/6 inhibitors alone^2–7^. Although high, the rate of grade 3–4 neutropenia we reported in the period between RT end and the three following cycles of CDK4/6 inhibitors (61.1%) is consistent with that described with palbociclib alone^3^. Moreover, no patient needed permanent suspension in the following cycles, with median duration of temporary interruption of 7 days and permanent dose reduction in only 14.3% of patients. Non-hematologic toxicity in our population was mild both in the acute and in the long term setting and represented mainly by grade 1 gastrointestinal toxicity. However, one of our patients also developed G3 ileitis 10 days after the end of RT with concurrent palbociclib on a bulky pelvic bone localization, that resolved completely after 20 days of conservative treatment. Another analogous case of severe radio-induced enterocolitis was reported after concomitant treatment with palbociclib and palliative radiotherapy (same dose, 30 Gy in 10 fractions) to left iliac bone and first sacral vertebra^13^. The patient developed worsening diarrhea and abdominal pain and a CT scan and colonoscopy confirmed the diagnosis. Following 3 weeks of conservative treatment, her symptoms abated. Biologically, 30 Gy in 10 fractions (EQD 2 Gy/fr 32.5 Gy) is a dose well below the normal bowel radiation tolerance dose, that is approximately 45–50 Gy in 2 Gy fractions^35^. Palbociclib might therefore act as a radiosensitizer on normal intestinal tissue, as palliative radiotherapy^36^ or palbociclib^2^ as single treatments are very unlikely to cause severe gastrointestinal side effects. In vivo data showed conflicting results, as palbociclib exposure resulted both in reduction^37,38^ and increase^38^ of acute gastrointestinal radiation syndrome in murine models, also depending on irradiation schedule^38^. Consequently, in case of concomitant CDK 4/6 inhibitor administration we advise the use of highly conformal treatment and optimization of the plan to maintain the dose as low as reasonably achievable to the gastrointestinal mucosa, especially in patients with high planning target volumes (PTVs) located in abdominal and pelvic areas and patients that experienced previous gastrointestinal toxicity. Only one patient (5.6%) experienced “pain flare” during radiotherapy, that resolved before the end of treatment with anti-inflammatory drugs: this compares favorably with the incidence reported in literature of up to 40% with RT alone^39^. In our population pain relief was complete in 8.3% and partial in 50% of patients at the last day of RT (median VNS 4 vs 5 at baseline), while the remaining 41.7% reported stable pain. One month after RT complete pain relief was experienced by 83.3% and after 3 months by 88.9% of patients and the remaining two patients obtained a VNS reduction of at least 50% compared to baseline. No pain recurrence was reported. Considering radiological and/or metabolic response, at three months 54.5% of patients achieved partial response and 45.5% stable disease and at 6 months 8.3% complete response, 75% partial response and 16.7% stable disease compared to baseline. With a median follow up of 13.7 months only one patient faced local recurrence while the other 94.4% of patients maintained local control. To our knowledge, this is the first report of concurrent treatment with radiation therapy and abemaciclib in metastatic breast cancer: in the three analyzed patients, no acute toxicity was observed.

The limits of our study must be acknowledged. Although our cohort, to the best of our knowledge, is the largest published up to date of metastatic breast cancer patients undergoing concurrent radiotherapy and CDK4/6 inhibitors, numbers are still limited. Results should therefore be interpreted with caution and should be confirmed by prospective studies enrolling larger samples of patients.

## Conclusions

Concomitant administration of palliative RT on bone lesions and CDK4/6 inhibitors is feasible and mostly characterized by a fair acute and long-term toxicity profile, with isolated episodes of high grade (although reversible) intestinal toxicity. Therefore, caution must be exercised when treating pelvic or abdominal sites, especially in case of high volume PTVs, and dose to the intestinal mucosa should be kept as low as reasonably achievable. In such scenarios, the use of highly conformal techniques is suggested. Rate of grade 3–4 neutropenia os high, but comparable with that measured for CDK4/6 inhibitors alone. Although the results of this study should be interpreted with caution, given the limited cohort,promising outcomes were reported both in terms of pain relief and local control of disease.
